# What factors influence couples’ decisions to have children? Evidence from a systematic scoping review

**DOI:** 10.1186/s12884-024-06385-3

**Published:** 2024-03-27

**Authors:** Mohammad Ranjbar, Mohammad Kazem Rahimi, Edris Heidari, Sajjad Bahariniya, Maliheh Alimondegari, Mohammad Hasan Lotfi, Tahereh Shafaghat

**Affiliations:** 1 Health Policy and Management Research Center, Department of Management and Health Economics Sciences, School of Public Health, Shahid Sadoughi University of Medical Sciences, Yazd, Iran; 2 Department of Health Services Management, School of Health Management and Information Sciences, Iran University of Medical Sciences, Tehran, Iran; 3 Department of Social Sciences, Yazd University, Yazd, Iran; 4 Department of Biostatistics and Epidemiology, School of Public Health, Spritual Health Research Center, Shahid Sadoughi University of Medical Sciences, Yazd, Iran

**Keywords:** Desire, Preference, Decision, Fertility, Childbearing, Couple, Family

## Abstract

**Background:**

One of the most significant demographic challenges over the past three decades has been the substantial reduction in fertility rates, worldwidely. As a developing country, Iran has also experienced a rapid decline in fertility over the past decades. Understanding factors influencing fertility is essential for development programs. Moreover, it’s crucial to study the parameters that affect the intention for childbearing in any society. Therefore, through a systematic scoping review, the present study investigates the factors influencing couples’ decisions toward childbearing.

**Methods:**

This study was a systematic scoping review conducted in 2023. To design and conduct this scoping review, Joanna Briggs Institute’s Protocol (Institute TJB, The Joanna Briggs Institute Reviewers ’ manual 2015; methodology for JBI scoping reviews, 2015) was used and the framework presented by Levac et al. (2010) was also used as a guide for conducting this review. Studies were searched in three main databases including ISI Web of Sciences, PubMed, and Scopus, using a predefined search strategy. Google Scholar was also used for complementary search. The search period was from 2002 to 2022.

**Results:**

A total of 18,454 studies were identified across three primary databases. After evaluating articles in three distinct phases based on title, abstract, and full-text, 46 articles were deemed eligible for inclusion in the scoping review. The qualitative analysis of the collected data from the selected studies through the scoping review led to classifying factors influencing households’ desire for childbearing into eight main themes and 101 sub-themes. The main themes associated with factors impacting households’ intention for childbearing encompass individual determinants, demographic and familial influencing factors, cultural elements, social factors, health-related aspects, economic considerations, insurance-related variables, and government support/incentive policies.

**Conclusions:**

Comprehensive and holistic attention from governments and officials toward the various factors affecting households’ intention and behavior regarding childbearing appears beneficial and effective. Furthermore, given the relative ineffectiveness of some of the current government’s supportive/incentive policies to increase couples’ desire for childbearing, it seems necessary to review and amend these policies. This review should address the most significant challenges and factors contributing to couples’ reluctance to childbearing or strengthen factors that can play a substantial role in fostering fertility and childbearing desires.

## Background

Population and associated issues are intricate and multidimensional topics within human societies [1]. The population plays a pivotal role in any society’s economic and social dynamics, and governments must skillfully manage fertility rates to attain sustainable development effectively [2]. Fertility, a significant natural phenomenon in every society, is a vital determinant of population growth, prompting nations to devise and implement incentivizing policies to augment it [3].

Over the past three decades, one of the foremost global demographic challenges has been the considerable decline in fertility rates [4]. Across the globe, numerous families are deferring childbirth for diverse reasons, potentially leading to a decrease in desired fertility rates, prolonged population decline, and ultimately a reduction in sustainable development. Furthermore, the proportion of those opting to remain childless is rising [5, 6]. These trends have resulted in declining fertility rates in many regions worldwide, especially in developing countries, contributing to an aging population, elevated retirement rates, a dearth of human resources for employment, and diminished economic growth and productivity [5].

Understanding the influential factors on fertility has become an indispensable prerequisite for development programs, particularly for developing countries with a heightened focus on economic progress [7]. The global fertility rate has decreased from 4.5 births per woman during 1970-1975 to 2.5 during 2005-2010 [4]. As a developing nation, Iran has also experienced a rapid decline in fertility over the past decade [8]. The fertility rate in Iran has similarly dwindled in recent years, reaching 1.9 between 1978 and 2015 [9]. Confronted with this dwindling population growth, the Iranian government initiated incentivizing policies in 2012. These policies encompassed designating homemaking as an occupation with a stipulated income, implementing schemes such as “Monthly Child Incentives,” “Extended Maternity Leave,” “Mandatory Health Insurance Coverage for Mothers and Children,” “Monthly Allowance for Unemployed Women with Young Children,” and “Free Pregnancy Food Basket” [10].

Numerous factors typically influence the decision to have children. However, under the sway of incentivizing policies, Iranian families displayed a reduced inclination to have more children, leading to a cessation in population growth and a decline in fertility [10, 11]. Nevertheless, a study by Hashemzadeh and colleagues (2022) demonstrated that effectively implemented family policies can positively influence the intention of young couples to have children. These policies encompass creating a conducive environment, facilitating work-life balance for couples, executing health promotion programs, offering child-centered social support, and enhancing social and cultural ties [12].

Families’ proclivity toward childbearing is shaped by a range of factors, including societal norms, economic circumstances, cultural considerations, personal beliefs, religion, partner attributes, educational levels, and economic uncertainties such as unemployment or job opportunities, work-related stress, delayed marriage, challenging housing conditions, and similar challenges [4, 13–15]. It could be argued that the desire for fertility in Iran is deeply intertwined with tradition, gender roles, and religious sentiments. The factors that underpin the desire for childbearing within families can ultimately lead to demographic shifts at the national level over time [9].

Parallel studies have explored the influential factors on couples’ inclination toward parenthood. In a study conducted in South Korea by Lee and Hwang (2017), the determinants of married women’s desire for parenthood were investigated. The study revealed that age, income level, and shared childcare responsibilities with spouses were pivotal factors influencing the intent for parenthood among working married women. Additionally, the findings underscored that government support for childcare significantly influences the future childbirth intentions of employed married women [13].

In another study, Rahman and colleagues (2020) identified the pivotal factors influencing fertility in developing nations. Their findings indicated that a woman’s age in developing countries significantly explains fertility outcomes. Increased education for spouses and women is associated with reduced fertility following age. Another unconventional factor influencing fertility behavior is a country’s per capita healthcare expenditure. Escalating healthcare costs per capita ultimately result in decreased fertility [16].

Similarly, a study by Soederberg and colleagues (2015) scrutinized women’s attitudes toward fertility and parenthood in Sweden. Their conclusions highlighted that age, occupation, residential area, and marital status impact attitudes toward fertility and parenthood. In light of individual differences and age, fostering awareness, dialogue, and necessary interventions in sexual and reproductive healthcare is imperative for addressing fertility concerns [17].

While couples’ inclination toward parenthood has consistently been a focal point of research, examining the influential variables on the intent for parenthood within a given society while considering its distinctive social, economic, and cultural contexts becomes highly imperative. Furthermore, grasping the factors that affect the desire and intent for fertility and parenthood through a comprehensive and all-encompassing approach can pave the way for more informed decision-making in crafting policies and initiatives to foster an increase in households’ willingness for fertility.

Consequently, the present study aims to comprehensively explore the factors influencing couples’ inclination toward parenthood through a systematic and organized review within this field.

## Methods

This study was a systematic scoping review conducted in 2022. To design and conduct this scoping review, Joanna Briggs Institute’s Protocol (2015) was used [18] and the framework presented by Levac et al. [19] was also used as a guide for conducting this review [20]. The scoping review, based on the framework presented by Levac et al. and the Joanna Briggs Institute’s Protocol, consists of 6 main stages, each of which is mentioned below:

1. Defining the research question and relating it to the study objective.

In this stage, according to the mentioned guidelines, the main research question was defined as follows: “What are the factors affecting the desire of couples to have children?” A clear research question in a scoping review serves as a guide for determining and selecting the criteria for including studies in the review process. Additionally, this clarity helps in designing a better study protocol, facilitating the search for evidence and studies, and creating a suitable structure for reporting the scoping review. The scoping review question should include the population, concept, and context (PCC) components [20].

In this study, the population (P) included all studies that studies factors affecting the desire of couples to have children. The concept (C) included the childbearing and desire to having children, and the context (C) included all countries that the couples live there.

2. Searching for relevant studies.

In this stage, studies were searched in three main databases including ISI Web of Sciences, PubMed, and Scopus, using a predefined search strategy. Google Scholar was also used for complementary search. The search period was from 2002 to 2022. Table 1 shows the search strategy used for searching relevant studies in this scoping review.

**Table 1:** The search strategy of the study

Databases		ISI web of science, PubMed, Scopus, Google Scholar
Search limitations		Language (English), In Title, Full text Available, Document type: Article, Review, Dissertation & Thesis
Search time-frame		2002.01.01 up to 2022.08.05
Search time		2022.07.05 up to 2022.08.05
Search terms	#1	Childbearing OR fertility OR "Reproductive Behavior" OR "Voluntary Childlessness" OR "Delayed Childbearing"
	#2	"factor*" OR "attitude*" OR "preference*" OR "desire*" OR "decision*" OR " decision*making"
Search strategy		#1 AND #2
Search strategy in PubMed		(((("Delayed Childbearing"[MeSH] OR "Reproductive Behavior"[MeSH] OR "Voluntary Childlessness"[MeSH] OR childbearing[Title] OR fertility[Title]) AND ("factor*"[Title] OR "attitude*"[Title] OR "preference*"[Title] OR "desire*"[Title] OR "decision*"[Title] OR "decision*making"[Mesh])) AND ("journal article"[Publication Type] OR review[Publication Type])) AND (English[Language])) AND (("2002"[Date - Publication] : "2022"[Date - Publication]))
Search strategy in ISI web of sciences		((((TI=(Childbearing OR fertility OR "Reproductive Behavior" OR "Voluntary Childlessness" OR "Delayed Childbearing")) AND TI=("factor*" OR "attitude*" OR "preference*" OR "desire*" OR "decision*" OR " decision*making")) AND DOP=(2002/2022)) AND DT=(Article OR Review)) AND LA=(English)
Search strategy in Scopus		( TITLE ( childbearing OR fertility OR "Reproductive Behavior" OR "Voluntary Childlessness" OR "Delayed Childbearing" ) AND TITLE ( "factor*" OR "attitude*" OR "preference*" OR "desire*" OR "decision*" OR " decision*making" ) AND LANGUAGE ( english ) ) AND PUBYEAR > 2001 AND PUBYEAR < 2023 AND ( LIMIT-TO ( DOCTYPE , "ar" ) OR LIMIT-TO ( DOCTYPE , "re" ) ) AND ( LIMIT-TO ( LANGUAGE , "English" ) ) AND ( LIMIT-TO ( SRCTYPE , "j" ) )

3. Screening and Selecting the Studies

The inclusion criteria for the studies were those that were in the form of original articles, reviews, or dissertations in English language, with full-text and addressed the factors affecting the couple’s desire for childbearing. The exclusion criteria were studies that lacked full-text or had non-English language or were book reviews, opinion articles, commentaries, letters to editors, or proceedings, which lacked the necessary framework for examining the specifications and evaluating the quality of the study.

In this stage, an attempt was made to access gray literature or studies that were not found in the search process by conducting a complementary search on Google Scholar and reviewing the references of the obtained studies. After searching the desired databases and removing duplicates, the studies were independently screened and reviewed by two members of the research team based on their title, abstract, and full-text in three phases. In each phase, the final decision on selecting the studies was made based on the agreement of both individuals, and in cases of disagreement, the opinion of the third member of the research team was taken.

**Fig. 1:**
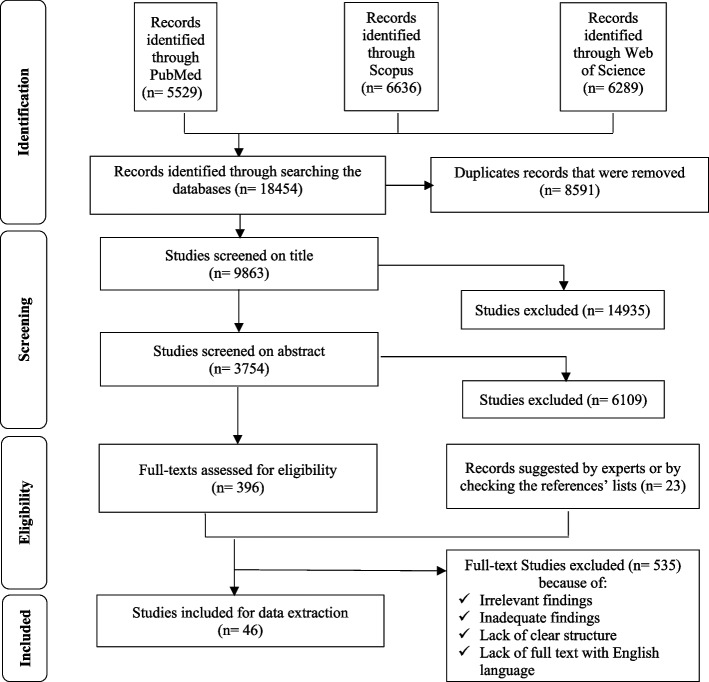
The PRISMA flow diagram for articles’ selection

Endnote software 8^th^ version was used to manage the process of systematic search and screening of studies. Moreover, the PRISMA protocol [21] was used to manage the process of selecting relevant studies and reporting this review (Fig. 1).

4. Analysis and charting the data

The process of extracting data from a scoping review is called charting the results. This process provides the reader a logical and concise summary of findings that is consistent with the objectives and questions of the scoping review. Create a charting table or form to record key source information such as authors, references, results, or findings related to the review question. Some important pieces of information that reviewers can choose to chart include author, year of publication, country of origin (published or research conducted), purpose/objectives, study population and sample size (if applicable), method, type of intervention, comparison and details (eg duration of intervention) (if applicable), intervention period (if applicable), results and related information (eg actions taken) (if applicable), and key findings related to scoping review questions. For ease of reference and tracking, reviewers are encouraged to keep careful records to identify each source [18, 20].

Therefore in present scoping review, after selecting the final studies based on the desired inclusion and exclusion criteria, data related to the factors affecting the couples’ desire to have children were extracted from the studies and entered into a study specifications table resulting from the scoping review. This table included the name of the first author, year of publication, study title, place of implementation, study design, participants, and key findings of the study. These results are presented in Table 2. Although the quality appraisal of the studies is not mandatory in the scoping review [22], the quality of the final selected studies according to the review was evaluated using specific quality assessment tools based on the each study’s type and method (Table 3). The studies obtained from the scoping review were assessed for quality using three established tools: the Critical Appraisal Skills Program (CASP) for qualitative research, the Appraisal Tool for Cross-Sectional Studies (AXIS tool), and the JBI Critical Appraisal Checklist for systematic reviews and research syntheses.

**Table 2:** The characteristic of the selected studies through systematic search

**Row**	**First author's name (year)**	**Study title**	**Place**	**Study design**	**Setting/ participants**	**Findings**	**Quality appraisal status**
**1**	Hosseinpoor, M. 2016 [10]	An Analysis on Views of Iranian Women about Incentive Policies on Childbearing Decision-making	Iran	descriptive-analytic cross-sectional Survey	stratified sampling 662 women between the ages of 15 and 49 years	□ Considering pension insurance for housewives □ Women are more inclined than men □ Family income rate □ Number of children	11/20
**2**	Li, X. H. 2019 [1]	Application of the Theory of Planned Behavior to couples’ fertility decision-making in Inner Mongolia, China	China	facility-based cross-sectional survey	sample size required for this study was 1,305	□ Attitudes: that only healthy parents can have children □ Adequate arrangements to support maternity and parental leave □ Perceived behavior control (the perceived importance of having a fixed income and the cost of raising children) □ Subjective norms (perceived social pressure regarding the baby’s sexual preference by themselves and their partners)	16/20
**3**	Hashemzadeh, M. 2021 [8]	Childbearing intention and its associated factors: A systematic review	Iran	A systematic review	none	□ Ecological factors in the following four levels: □ Macro system: culture, family policies, religiosity, child value, teachings of individualism □ Microsystem: socio-demographic characteristics (age, education, financial status), physical and mental health, happiness (desire of the child) □ Mesosystem: marital status, equality, participation satisfaction, gender role awareness, family and peer network □ Exo system: job characteristics and urban residence, housing situation	6/11
**4**	Adhikari, R. 2010 [23]	Demographic, socio-economic, and cultural factors affecting fertility differentials in Nepal	Nepalese	nationally cross-sectional Survey	Nepal Demographic and Health Survey (NDHS) married women of reproductive age (8,644)	□ Age at first marriage □ Perceived ideal number of children □ Mass media exposure □ Literacy status	14/20
**5**	Chen, M. N. 2017 [24]	The Discrepancy Between Ideal and Actual Parity in Hong Kong: Fertility Desire, Intention, and Behavior	Hong Kong	cross-sectional Survey	married or cohabiting women aged 15–49 and their spouses. 1029 couples	□ Satisfaction with married life □ Household income □ Good communication with husbands □ Good income and healthy marriage are prerequisites for having children	15/20
**6**	Khedmat, L. 2022 [9]	Factors affecting childbearing decision making among Iranian couples: a qualitative study	Iran	qualitative study	15 Iranian couples using a conventional content analysis	□ Factors affecting the intention to have children into four main categories: □ Ability of couples □ Parental attitudes □ Couple interactions □ Childbearing experiences	7/9
**7**	Sabermahani, A. 2017 [2]	Factors Affecting Fertility Rate in Iran (Panel Data 1966-2013): A Survey Study	Iran	descriptive-analytic study combination of cross-sectional and time series data	data were extracted from all residents of country differentiated by 24 and from statistical yearbooks of Statistical Center of Iran	□ Creating conditions for marriage by reducing unemployment □ Marriage is the main and most important factor affecting the fertility rate □ Educated women emphasize more on the quality of their children than their quantity	14/20
**8**	Aghoozi, M. F. 2020 [25]	Factors Affecting the First Childbearing Decision in Iranian Males	Iran	analytical cross-sectional study	300 married men aged 18-45 years	□ Marriage age of men □ Quality of life	16/20
**9**	Moradi, M. 2017 [26]	The factors associated with childbearing intentions in Iranian female University students	Iran	cross-sectional study	randomly examined 294 female students of Shahid Beheshti University	□ Personal factors □ Family factors □ The role of the wife □ Perceived social support and financial factors □ Beliefs	16/20
**10**	Lee, M. J. 2017 [13]	Factors contributing to childbearing intentions of married working women in Korea	Korea	cross-sectional study	sing the 2012 National Survey a total of 1,408 respondents were selected based on marriage, work and age	□ Gender equality and shared child care with spouse □ Income level □ Government support for child care and support at work	15/20
**11**	Araban, M. 2020 [4]	Factors related to childbearing intentions among women: a cross-sectional study in health centers, Saveh, Iran	Iran	A cross-sectional study	A total of 483 married women 15–49 years old participated in this study	□ Psychological factors such as marital satisfaction □ Social support □ Attitude □ Social norms □ Hope, perceived social support and marital satisfaction	17/20
**12**	Lui, L. K. 2021 [3]	Family policies, social norms and marital fertility decisions: A quasi-experimental study	Hong Kong	household survey quasi-experimental design	sample size of 1,000 married adults aged under 40	□ Policies that reduce time constraints between work and family □ Paid/unpaid parental leave law and working hours) □ Financial expenses (providing child care, allowance and housing allowance)	16/20
**13**	Karabchuk, T. 2022 [5]	Fertility attitudes of highly educated youth: A factorial survey	five countries	A factorial survey	Data were collected from Germany, Japan, Russia, Ukraine, and the United Arab Emirates (UAE)	□ Income □ Availability of childcare □ Full employment of the husband □ Child friendly policies	14/20
**14**	Kan, M. Y. 2019 [27]	Housework share and fertility preference in four East Asian countries in 2006 and 2012	Japan, South Korea, and Taiwan	2006 East Asian Social Survey (EASS) and the 2012 International Social Survey Program	married couples where women were over 20 and under 45 years of age (*N* = 6,410: China *n* = 3,635, Japan *n* = 736, Korea *n* = 928, Taiwan *n* = 1,111).	□ Gender equal division of household labor in East Asia is associated with higher fertility intentions in this region □ The number of ideal children of women whose husbands participate in housework is more	13/20
**15**	Rosniza Aznie, C. R. 2013 [14]	Identifying potential factors of ideal childbearing among Malay women in Terengganu	Malaysia	survey	ample size was 348	□ Age of first marriage □ Health factors □ Type of work	11/20
**16**	Rahman, A. 2020 [16]	Influencing Factors of Fertility in Developing Countries: Evidence from 16 DHS Data	16 different countries’	demographic and health survey data	women residing in developing countries	□ Women’s age and age at first marriage □ Education □ Urbanization leads to decrease in fertility □ Women’s wealth index □ Body mass index	15/20
**17**	Soltanian, A. 2019 [11]	Modeling the Factors Affecting the First Birth in the Family’s’ Fertility in Hamedan Province	Iran	cross-sectional (descriptive-analytic) study	married woman in the age group of 15 to 49 years in 500 families in Hamedan	□ Parent education □ Employment of women □ Use of preventive measures □ Raising the level of community awareness	16/20
**18**	Bagheri, A. 2019 [28]	Modelling Childbearing Desire: Comparison of Logistic Regression and Classification Tree Approaches	Iran	survey	4898 women for childbearing in provinces with a TFR lower	□ Adopting policies to change women’s views on childbearing □ Creating the necessary resources to avoid delay in marriage □ Their opinion about having children	16/20
**19**	Wei, J. Q. 2018 [7]	Socioeconomic determinants of rural women’s desired fertility: A survey in rural Shaanxi, China	China	cross-sectional survey	The targeted population was married women of childbearing age (20–49) with a rural household identification 2,516	□ Cultural view of rural women towards fertility □ Major economic factors including social security coverage for the elderly □ Direct costs of having a child	18/20
**20**	Soderberg, M. 2015 [17]	Women’s attitudes towards fertility and childbearing - A study based on a national sample of Swedish women validating the Attitudes to Fertility and Childbearing Scale (AFCS)	Sweden	A study based on a national sample of Swedish women validating the Attitudes to Fertility and Childbearing Scale	women in Sweden who are 20–30 years Four hundred and twenty-four women, 20–30 years	□ Age □ Job □ Residential area □ Marital status	16/20
**21**	Kariman, N. 2016 [29]	A Path Analysis of Factors Influencing the First Childbearing Decision-Making in Women in Shahroud in 2014	Iran	cross-sectional study	y was conducted on 300 eligible pregnant women admitted to healthcare centers	□ Individual factors (marriage age, hope and quality of life) □ Family factors (marital satisfaction) □ Social factors (social support) □ Marriage age, marital satisfaction, social support, economic status, hope and quality of life	18/20
**22**	Hashemzadeh, M. 2022 [12]	Principal factors affecting couples’ childbearing policies: A roadmap for policymaking	Iran	descriptive cross-sectional study	300 couples Of the 360 questionnaires, 60 incomplete forms were removed and 300 filled questionnaires were considered	□ Family policies include: □ Supporting couples to integrate work and home □ Health promotion programs □ Social support of missing child □ Improving the level of social and cultural relations	16/20
**23**	Kiani, M. 2011 [30]	Women’s attitude to fertility in Iran: A case study in Isfahan, Iran	Iran	survey	300 people were studied women are 20–45 years	□ Length of married life □ Male marriage age □ Job □ Education	11/20
**24**	Ahinkorah, B. O. 2021 [15]	Which factors predict fertility intentions of married men and women? Results from the 2012 Niger Demographic and Health Survey	Niger Demographic and Health Survey	survey	total of 2,186 childbearing men and 5,969 childbearing women aged 25–59 and 25–49 respectively adolescents and young women aged 15–24 were excluded.	□ Socio-economic and demographic factors □ Prevalence of desire for fertility is higher among men than women	15/20
**25**	Boivin, J. 2018 [31]	What makes people ready to conceive? Findings from the International Fertility Decision-Making Study	79 countries	cross-sectional survey	10,045 participants (1690 men and 8355 women) from 79 countries. Respondents were aged 18-50 years (mean 31.8 years).	□ Women had a higher personal desire to have children and evaluated economic, personal and relational preparation as more effective □ Males were more likely to evaluate the mental norms and social status of parents as more influential □ Personal desire for the child □ Desire of the partner for the child □ Need for parents □ Motivational forces and mental norms	18/20
**26**	Evens, E. 2015 [6]	Identifying factors that influence pregnancy intentions: evidence from South Africa and Malawi	South Africa and Malawi	qualitative analysis	Data from a total of 199 women is included here, 113 in FGDs and 86 in SSIs	□ Social norms during pregnancy □ HIV related concerns □ Partnership status and relationship quality □ Available financial resources □ Demographic characteristics □ Mother’s attitude and experiences towards pregnancy	8/9
**27**	Holton, S. 2011 [32]	To have or not to have? Australian women’s childbearing desires, expectations and outcomes	Australia	a cross-sectional survey	569 30–34-years-old Australian women randomly selected	□ Various biological, psychological and social factors □ Costs of women having children	16/20
**28**	Chen, P. L. 2021 [33]	A new model for evaluating the influence of social networks, social learning, and supportive policies on the desire of women for fertility	Iran	cross- sectional	From 384 users of Telegram data are collected	□ Social networks are meaningful and positive on social learning □ The role of social learning and supportive policies on women’s desire for positive fertility	15/20
**29**	Pan, J. N. 2020 [34]	The impact of economic uncertainty on the decision of fertility: Evidence from Taiwan	Taiwan	cross- sectional	official county-level panel data of 20 counties and cities over the 1998–2016 period in Taiwan Family Income and Expenditure (1998–2016) published by the Directorate General of Budget, Accounting and Statistics (DGBAS)	□ Economic uncertainty □ Higher social welfare costs □ Comprehensive maternity subsidy policy □ Fluctuations in household disposable income □ Unemployment rate and aging	8/20
**30**	Kaboudi, Marzieh 2013 [35]	The decision-making process of childbearing: a qualitative study	Iran	quantitative methods	In-depth interviews were carried out with 20 married women and 14 married men. 34	□ Understanding the amount of control the mother has over the conditions after childbirth	6/9
**31**	Samani, Leila 2020 [36]	The Influence of Legal Supports of Working Women during Pregnancy and Lactation Period on Their Desire to Have Children	Iran	correlational-descriptive	Working women of Fasa Medical Society women 80	□ Legal protection of the government for working women □ Employment conditions □ Organizational facilities and maternity leave	6/20
**32**	Dorahaki, Ahmad 2021 [37]	Explanation of Psychosocial Factors Affecting Fertility Behavior: Study of Fertility Behavior Among Married Women Aged 15 to 49 in Nasimshar	Iran	cross- sectional	Data were collected based on surveys on 304 married women aged 15 to 49 in Nasimshahr. Sample is selected whit multistage cluster.	□ Lowering the age of marriage □ Improvement of economic conditions □ Reducing the cost of living for households □ Emotional need for the child	15/20
**33**	Begi, Milad 2022 [38]	Desire to childbearing in Iran: determinants and barriers	Iran	Survey	Married women of reproductive age (15-49 years old) and men whose wives are of reproductive age. Finally, information related to 2118 people living in urban areas of 31 provinces of the country was analyzed	□ Religious beliefs □ Satisfaction with life and marital relationship	12/20
**34**	Haerimehrizi, Ali Asghar 2017 [39]	Reasons for fertility desire and disinterest among Iranian married adults: A population-based study	Iran	cross- sectional population -based study	A total of 20,935 married citizens from all over Iran In all 20935 individuals (10388 male and 10547 female)	□ Parents’ interest in having children □ Strengthening social economic infrastructure	8/20
**35**	Bagheri, A. 2017 [40]	Identification of fertility preferences determinants using poisson regression	Iran	Cross-sectional survey	In 2012, 389 ever married women aged 15-49 with two-stage stratified sampling method	□ Number of children born alive □ job status (employment) □ Education level □ Type of marriage □ Residence	13/20
**36**	Alidousti, Ezddin 2021 [41]	Socio-economic factors affecting attitudes towards childbearing: A study of ever married couples in Kermanshah, Iran	Iran	Survey	The sample comprised of 374 ever married men and women aged 15 -49 in Kermanshah, Iran. clustered sampling	□ Gender preference □ Ideal age gap between children □ Duration of Internet use □ Distance from marriage to first child	13/20
**37**	Rahnama, Ameneh 2022 [42]	Factors Related to Childbearing in Iran: A Systematic Review	Iran	A Systematic Review	A Systematic Review	□ Employment status and job grade □ Marriage age □ Education level □ Social economic conditions □ having a religion □ Family orientation	5/11
**38**	Abbasi, Amene 2022 [43]	A Meta-Analysis of Factors Related to Fertility Attitudes , Desires , and Childbearing Intentions in Iranian Studies	Iran	A Meta-Analysis	6 articles have been reviewed between 1389 and 1398. 16 articles have investigated the fruitfulness and 10 articles have investigated the attitudes of students.	□ Age □ Rural residence □ Income □ having a religion □ Social support □ Number of siblings □ Quality of life	9/11
**39**	Tavousi, Mahmoud 2016 [44]	Fertility desire among Iranians living in Tehran: reasons for desire and disintere	Iran	population -based study	A sample of married individuals living in all 22 districts in Tehran : In all 1200 individuals (600 male and 600 female)	□ The natural interest of people to acquire the status and role of parents □ Promoting a positive experience of parenthood	11/20
**40**	Torkian Valashani, Sahebjan 2019 [45]	Fertility desire: Facilitators and inhibitors	Iran	cross -sectional study	A total of 400 married individuals were entered into the study A simple random image is available	□ Lowering the cost of infertility treatment □ Belief in the role of the child in strengthening the family □ Belief in the usefulness of children in old age	11/20
**41**	Ayazi, Rozita (2021) [46]	Factors Related to Childbearing Willingness in the Women Attending the Health Centers in Arak, Iran (2019)	Iran	cross-sectional study	255 women aged 18-45 years from 10 health centers in Arak city	a direct relationship can be concluded between the variables of social participation, economic status, marital satisfaction, childbearing problems with childbearing unwillingness in women referring to Arak health centers at 95% confidence interval.	
**42**	Erfani, Amir (2019) [47]	Reasons for intending to have no children in Tehran, Iran	Iran	cross -sectional study (interviewing respondents face-to-face with a structured questionnaire)	married men and women under aged 36 living in 22 districts of the city of Tehran ( n = 2267)	the most important reasons for not wanting any [48] children: □ “Not being able to afford the cost of raising children” (27%), □ “Having the desired number of children” (25%), □ “being worry about the future of their children” (15%) as □ “conflict of childbearing with own personal life, plans and interests” (16%), □ “spouse’s opposition” (6%) □ “problems in spousal relationships” (2%)	
**43**	Gaffari, Fatemeh (2021) [49]	Factors Affecting Childbearing Based On Women’s Perspectives: A Qualitative Study	Iran	Qualitative Study (semi-structured interviews)	25 women of reproductive age from health centers in Mashhad	Negative factors on childbearing: □ economic problems (the most effective factor in childbearing) □ Fear of social insecurity □ Positive factors on childbearing: □ cultural factors such as the age of marriage □ religious factors such as trusting God with child’s aliment Also, the government’s incentive and restrictive policies to increase fertility are effective in childbearing	
**44**	Mobasheri, Mahmood (2013) [50]	Determination of the Most Important Factors Influencing the Fertility Patterns of Single Child and Without Child Families in Shahr-e-kord City in 2013	Iran	cross-sectional study	180 married women	□ There was a significant direct relationship between the score of attitude and age, age at the time of marriage, duration of marriage and education with childbearing desire. □ Causes of disinclined to childbearing were noted by the highest percentage of participants (83.3%) as increase in costs and economic pressures and by the lowest percentage of participants (8.3%) as fear of recurrent miscarriage and stillbirth.	
**45**	Nematian, Sareh (2021) [51]	The Impact of Couples’ Decision-Making Process in Delayed Childbearing and Related Social Conditions	Iran	Qualitative study; narrative interviews	eight couples (who are not willing to have child even a few years after their marriage)	□ Couples’ economic situation, social network, relationship with each other, as well as their personal purposes and motivation had a significant role in decision-making process about postponing childbearing.	
**46**	Vahdani, Fahimeh (2018) [52]	Fertility Style and its Determinants in Iran and Islamic Countries : A Review Study	Iran	Review Study	14 selected studies	□ Religious beliefs, the age at marriage and maternal age were positive factors in the fertility style. □ Low economic status, high literacy and cultural activities had a negative relationship with childbearing.

**Table 3:** The checklist of quality appraisal of the selected studies through systematic scoping review

Study title	**Critical Appraisal Skills Program (CASP) for assessing the quality of qualitative researches (**yes/no/can’t tell**)**										
	Was there a clear statement of the aims of the research?	Is a qualitative methodology appropriate?	Was the research design appropriate to address the aims of the research? (Appropriate research design)	Was the recruitment strategy appropriate to the aims of the research? (Sampling)	Were the data collected in a way that addressed the research issue? (Data collection)	Has the relationship between researcher and Participants been adequately considered? (Reflexivity)	Have ethical issues been taken into consideration? (Ethical Issues)	Was the data analysis sufficiently rigorous? (Data Analysis)	Is there a clear statement of findings? (Findings)	How valuable is the research? (Value of the research)	
Factors affecting childbearing decision making among Iranian couples: a qualitative study	yes	yes	yes	yes	yes	can’t tell	yes	can’t tell	yes	7/9	
Identifying factors that influence pregnancy intentions: evidence from South Africa and Malawi	yes	yes	yes	yes	yes	yes	can’t tell	yes	yes	8/9	
The decision-making process of childbearing: a qualitative study	yes	yes	can’t tell	yes	yes	no	yes	yes	can’t tell	6/9	
The Impact of Couples' Decision-Making Process in Delayed Childbearing and Related Social Conditions	yes	yes	yes	yes	yes	yes	can’t tell	yes	yes	8/9	
Factors Affecting Childbearing Based On Women's Perspectives: A Qualitative Study	yes	yes	yes	yes	yes	can’t tell	yes	can’t tell	yes	7/9	
Study title	**Appraisal tool for Cross-Sectional Studies (AXIS tool)** (yes/no/do not know)										
	**Introduction**	**Methods**									
	Were the aims/objectives of the study clear?	Was the study design appropriate for the stated aim(s)?	Was the sample size justified?	Was the target/reference population clearly defined? (Is it clear who the research was about?)	Was the sample frame taken from an appropriate population base so that it closely represented the target/reference population under investigation?	Was the selection process likely to select subjects/participants that were representative of the target/reference population under investigation?	Were measures undertaken to address and categorize non-responders?	Were the risk factor and outcome variables measured appropriate to the aims of the study?	Were the risk factor and outcome variables measured correctly using instruments/measurements that had been piloted or published previously?	Is it clear what was used to determined statistical significance and/or precision estimates? (eg, p values, CIs)	Were the methods (including statistical methods) sufficiently described to enable them to be repeated?
An Analysis on Views of Iranian Women about Incentive Policies on Childbearing Decision-making	yes	yes	yes	yes	yes	Do not know	no	no	yes	yes	Do not know
Application of the Theory of Planned Behavior to couples' fertility decision-making in Inner Mongolia, China	yes	yes	yes	yes	yes	yes	no	yes	do not know	yes	yes
Demographic, socio-economic, and cultural factors affecting fertility differentials in Nepal	yes	do not know	no	yes	yes	do not know	no	yes	yes	yes	yes
The Discrepancy Between Ideal and Actual Parity in Hong Kong: Fertility Desire, Intention, and Behavior	yes	yes	yes	yes	yes	yes	do not know	do not know	yes	yes	yes
Factors Affecting Fertility Rate in Iran (Panel Data 1966-2013): A Survey Study	yes	yes	no	do not know	yes	yes	no	yes	yes	yes	yes
Factors Affecting the First Childbearing Decision in Iranian Males	yes	yes	Yes	yes	yes	yes	no	yes	yes	yes	do not know
The factors associated with childbearing intentions in Iranian female University students	yes	yes	yes	yes	yes	yes	do not know	yes	yes	yes	yes
Factors contributing to childbearing intentions of married working women in Korea	yes	yes	yes	yes	yes	do not know	no	yes	yes	yes	yes
Factors related to childbearing intentions among women: a cross-sectional study in health centers, Saveh, Iran	yes	yes	yes	yes	yes	Yes	do not know	yes	yes	yes	yes
Family policies, social norms and marital fertility decisions: A quasi-experimental study	yes	do not know	Yes	yes	yes	do not know	no	yes	yes	yes	yes
Fertility attitudes of highly educated youth: A factorial survey	Yes	yes	do not know	yes	yes	yes	do not know	yes	yes	yes	yes
Housework share and fertility preference in four East Asian countries in 2006 and 2012	yes	yes	no	yes	do not know	yes	no	yes	yes	yes	yes
Identifying potential factors of ideal childbearing among Malay women in Terengganu	yes	do not know	yes	yes	do not know	yes	no	do not know	yes	yes	yes
Influencing Factors of Fertility in Developing Countries: Evidence from 16 DHS Data	yes	yes	yes	yes	Yes	do not know	no	yes	yes	yes	yes
Modeling the Factors Affecting the First Birth in the Family's' Fertility in Hamedan Province	yes	yes	no	yes	yes	no	yes	yes	yes	yes	yes
Modelling Childbearing Desire: Comparison of Logistic Regression and Classification Tree Approaches	yes	yes	yes	no	yes	yes	no	yes	yes	yes	yes
Socioeconomic determinants of rural women's desired fertility: A survey in rural Shaanxi, China	yes	yes	yes	yes	yes	yes	do not know	yes	yes	yes	yes
Women's attitudes towards fertility and childbearing - A study based on a national sample of Swedish women validating the Attitudes to Fertility and Childbearing Scale (AFCS)	yes	yes	yes	yes	yes	yes	No	do not know	yes	yes	yes
A Path Analysis of Factors Influencing the First Childbearing Decision-Making in Women in Shahroud in 2014	yes	yes	yes	yes	yes	yes	no	yes	yes	yes	yes
Principal factors affecting couples' childbearing policies: A roadmap for policymaking	yes	yes	yes	do not know	yes	do not know	no	yes	yes	yes	yes
Women's attitude to fertility in Iran: A case study in Isfahan, Iran	yes	yes	no	yes	yes	no	no	yes	yes	do not know	yes
Which factors predict fertility intentions of married men and women? Results from the 2012 Niger Demographic and Health Survey	yes	yes	do not know	yes	yes	yes	no	yes	yes	yes	do not know
What makes people ready to conceive? Findings from the International Fertility Decision-Making Study	yes	yes	yes	yes	yes	yes	no	yes	yes	yes	yes
To have or not to have? Australian women's childbearing desires, expectations and outcomes	yes	yes	yes	yes	yes	yes	no	yes	yes	do not know	yes
A new model for evaluating the influence of social networks, social learning, and supportive policies on the desire of women for fertility	yes	yes	yes	yes	do not know	yes	no	yes	yes	yes	yes
The impact of economic uncertainty on the decision of fertility: Evidence from Taiwan	yes	yes	do not know	yes	do not know	do not know	no	yes	yes	no	no
The Influence of Legal Supports of Working Women during Pregnancy and Lactation Period on Their Desire to Have Children	yes	do not know	yes	no	do not know	no	no	do not know	yes	no	do not know
Explanation of Psychosocial Factors Affecting Fertility Behavior: Study of Fertility Behavior Among Married Women Aged 15 to 49 in Nasimshar	yes	yes	yes	yes	yes	yes	no	yes	yes	no	yes
Desire to childbearing in Iran: determinants and barriers	yes	yes	no	yes	yes	do not know	no	yes	yes	do not know	yes
Reasons for fertility desire and disinterest among Iranian married adults: A population-based study	yes	do not know	no	yes	yes	do not know	no	yes	yes	no	do not know
Identification of fertility preferences determinants using poisson regression	yes	yes	no	yes	do not know	yes	no	yes	yes	yes	yes
Socio-economic factors affecting attitudes towards childbearing: A study of ever married couples in Kermanshah, Iran	yes	yes	no	yes	do not know	yes	no	yes	yes	yes	yes
Fertility desire among Iranians living in Tehran: reasons for desire and disinterest	yes	yes	yes	yes	do not know	do not know	no	yes	yes	yes	no
Fertility desire: Facilitators and inhibitors	yes	do not know	yes	yes	yes	do not know	no	yes	yes	no	yes
Study title	**Results**					**Discussion**		**Other**			
	Were the basic data adequately described?	Does the response rate raise concerns about non-response bias?	If appropriate, was information about non-responders described?	Were the results internally consistent?	Were the results for the analyses described in the methods, presented?	Were the authors’ discussions and conclusions justified by the results?	Were the limitations of the study discussed?	Were there any funding sources or conflicts of interest that may affect the authors’ interpretation of the results?	Was ethical approval or consent of participants attained?		
An Analysis on Views of Iranian Women about Incentive Policies on Childbearing Decision-making	yes	no	no	yes	yes	yes	do not know	no	no		
Application of the Theory of Planned Behavior to couples’ fertility decision-making in Inner Mongolia, China	yes	yes	no	yes	yes	yes	yes	do not know	yes		
Demographic, socio-economic, and cultural factors affecting fertility differentials in Nepal	yes	no	no	yes	yes	yes	yes	yes	yes		
The Discrepancy Between Ideal and Actual Parity in Hong Kong: Fertility Desire, Intention, and Behavior	yes	no	no	yes	yes	yes	yes	do not know	yes		
Factors Affecting Fertility Rate in Iran (Panel Data 1966-2013): A Survey Study	yes	do not know	no	yes	yes	yes	yes	yes	do not know		
Factors Affecting the First Childbearing Decision in Iranian Males	yes	do not know	no	yes	yes	yes	yes	yes	yes		
The factors associated with childbearing intentions in Iranian female University students	do not know	yes	no	yes	yes	yes	no	yes	yes		
Factors contributing to childbearing intentions of married working women in Korea	yes	yes	no	yes	yes	yes	do not know	no	yes		
Factors related to childbearing intentions among women: a cross-sectional study in health centers, Saveh, Iran	yes	yes	do not know	yes	yes	yes	yes	no	yes		
Family policies, social norms and marital fertility decisions: A quasi-experimental study	yes	yes	no	yes	yes	yes	yes	yes	yes		
Fertility attitudes of highly educated youth: A factorial survey	yes	no	no	yes	yes	yes	do not know	yes	do not know		
Housework share and fertility preference in four East Asian countries in 2006 and 2012	yes	no	no	yes	yes	yes	yes	no	no		
Identifying potential factors of ideal childbearing among Malay women in Terengganu	yes	no	no	yes	yes	yes	no	no	do not know		
Influencing Factors of Fertility in Developing Countries: Evidence from 16 DHS Data	yes	yes	no	yes	yes	yes	do not know	no	yes		
Modeling the Factors Affecting the First Birth in the Family’s’ Fertility in Hamedan Province	yes	no	no	yes	yes	yes	yes	yes	yes		
Modelling Childbearing Desire: Comparison of Logistic Regression and Classification Tree Approaches	yes	no	no	yes	yes	yes	yes	yes	yes		
Socioeconomic determinants of rural women’s desired fertility: A survey in rural Shaanxi, China	yes	yes	no	yes	yes	yes	yes	yes	yes		
Women’s attitudes towards fertility and childbearing - A study based on a national sample of Swedish women validating the Attitudes to Fertility and Childbearing Scale (AFCS)	yes	yes	no	yes	yes	yes	yes	no	yes		
A Path Analysis of Factors Influencing the First Childbearing Decision-Making in Women in Shahroud in 2014	yes	yes	no	yes	yes	yes	yes	yes	yes		
Principal factors affecting couples’ childbearing policies: A roadmap for policymaking	yes	yes	no	yes	yes	yes	yes	yes	yes		
Women’s attitude to fertility in Iran: A case study in Isfahan, Iran	yes	no	no	yes	yes	yes	no	no	do not know		
Which factors predict fertility intentions of married men and women? Results from the 2012 Niger Demographic and Health Survey	yes	no	no	yes	yes	yes	yes	yes	yes		
What makes people ready to conceive? Findings from the International Fertility Decision-Making Study	yes	yes	no	yes	yes	yes	yes	yes	yes		
To have or not to have? Australian women’s childbearing desires, expectations and outcomes	yes	yes	no	yes	yes	yes	yes	no	yes		
A new model for evaluating the influence of social networks, social learning, and supportive policies on the desire of women for fertility	yes	yes	no	yes	yes	yes	yes	no	no		
The impact of economic uncertainty on the decision of fertility: Evidence from Taiwan	yes	no	no	yes	do not know	yes	do not know	do not know	do not know		
The Influence of Legal Supports of Working Women during Pregnancy and Lactation Period on Their Desire to Have Children	do not know	no	no	yes	yes	yes	no	no	no		
Explanation of Psychosocial Factors Affecting Fertility Behavior: Study of Fertility Behavior Among Married Women Aged 15 to 49 in Nasimshar	yes	yes	no	yes	yes	yes	no	no	yes		
Desire to childbearing in Iran: determinants and barriers	yes	do not know	no	yes	yes	yes	no	no	yes		
Reasons for fertility desire and disinterest among Iranian married adults: A population-based study	yes	no	no	do not know	yes	yes	no	no	do not know		
Identification of fertility preferences determinants using poisson regression	yes	no	no	yes	yes	yes	no	yes	do not know		
Socio-economic factors affecting attitudes towards childbearing: A study of ever married couples in Kermanshah, Iran	yes	no	no	yes	yes	yes	yes	no	do not know		
Fertility desire among Iranians living in Tehran: reasons for desire and disintere	do not know	yes	no	yes	yes	yes	no	no	no		
Fertility desire: Facilitators and inhibitors	no	do not know	no	yes	yes	yes	yes	no	no		
Study title	**JBI Critical Appraisal Checklist for systematic reviews and research syntheses** (Yes/ No/ Unclear/ Not applicable)										
	Is the review question clearly and explicitly stated?	Were the inclusion criteria appropriate for the review question?	Was the search strategy appropriate?	Were the sources and resources used to search for studies adequate?	Were the criteria for appraising studies appropriate?	Was critical appraisal conducted by two or more reviewers independently?	Were there methods to minimize errors in data extraction?	Were the methods used to combine studies appropriate?	Was the likelihood of publication bias assessed?	Were recommendations for policy and/or practice supported by the reported data?	Were the specific directives for new research appropriate?
Childbearing intention and its associated factors: A systematic review	Unclear	yes	Unclear	yes	Unclear	no	yes	yes	Unclear	yes	yes
Factors Related to Childbearing in Iran: A Systematic Review	no	yes	Unclear	yes	no	no	Unclear	yes	Unclear	yes	yes
A Meta-Analysis of Factors Related to Fertility Attitudes, Desires , and Childbearing Intentions in Iranian Studies	yes	yes	yes	yes	Unclear	Unclear	yes	yes	yes	yes	yes
Fertility Style and its Determinants in Iran and Islamic Countries : A Review Study	yes	yes	yes	yes	Unclear	Unclear	yes	yes	yes	yes	yes

Subsequently, the collected data underwent iterative qualitative analysis to ensure comprehensive coverage of complex topics and concepts relevant to the review’s context. This method facilitated the identification and summarization of various aspects concerning factors influencing couples’ desire to have children and enabled accurate interpretation of the study texts. Unforeseen data discovered during interpretation were continuously incorporated into the evolving results, with the graphical table being regularly updated. It is advisable for the review team to familiarize themselves with the original results and test the data extraction format in a few studies to ensure comprehensive extraction. This approach is commonly favored by practitioners conducting scoping reviews [18].

5. Summarizing and reporting

The summary of findings elucidates the objectives of the included articles, the underlying concepts and approaches, and the results pertaining to the review questions. Extracted results were categorized into broader theoretical groupings aligned with the review’s focus [20]. In this stage, two authors independently screened and reviewed the text and results of each selected study in the review and extracted related meaningful codes according to the review purpose. Then they integrated and summarized the data obtained from the studies’ texts to reach the sub-themes related to the factors affecting the couples’ desire to have children. In cases of possible disagreements, a third person in research team would help to reach a consensus. Finally, the main themes related to the topic were defined, clarified, and categorized as a comprehensive set of factors affecting the couples’ desire to have children (Table 4).

**Table 4:** Factors influencing couples’ childbearing preferences

**Themes**	**Sub-themes**	**Related codes**
**Personal and Family Context**	**Demographic Factors**	Age of Spouses [4, 7, 8, 11, 13, 14, 16, 17, 28, 37–39, 41–45, 50, 52]
		Age of Spouses at the Time of Marriage [7, 11, 12, 23, 29, 30, 37, 42, 43, 49, 50, 52]
		Educational Level of Spouses [4, 8, 12, 14, 16, 23, 37, 38, 40, 42, 43, 50, 52]
		Duration of Married Life [30, 50]
		Gender Preference [1, 37, 38, 41]
		Number of current children [6, 10–12, 15, 23, 28, 37–40, 42–45, 47, 49]
	**Family Dynamics and Relationships**	The desire to have a large family [37, 39, 43–45] and Possibility of generational continuity [35]
		Polygamy [49]
		Intervals Between Births [11, 41]
		Challenges of Child Rearing [46]
		Spouse’s opinions and expectations Regarding Parenthood [6, 38, 41, 44, 47, 49–51]
		Motivation and Hope for Life and the Future [4, 29, 38, 42]
		Satisfaction with Married Life [4, 8, 24, 25, 29, 31, 38, 42, 43, 46, 47] and Good Spousal Communication [9, 24, 35, 38]
		Commitment to Life and Family [42] and Strengthening family relationships [35, 39, 45, 49]
		Spousal Attitudes Towards Fertility and their Inherent Interest in Children and Attaining a Parental Role [4, 9, 35, 37–39, 42, 44, 45]
		Lack of Adequate Time [35, 37, 50]
		Experiencing Non-Spousal Marriage [40]
		Previous Experiences of Childbearing/Birth or Child Mortality [9, 11, 41, 49]
		Fear of Childbirth/Miscarriage and Stillbirth [50]
		Parental support in childcare [8, 13, 26, 27, 31, 35]
		Conflict of Child Rearing with Individual’s Interests, Leisure Activities, Work, and Educational Programs [38, 42, 44, 45, 47, 50]
		Concerns About the Future of Children [35, 38, 39, 42, 44, 45, 47]
		Supporting children in their elderly years [35, 37, 45]
	**Health and Well-being**	Perceived Ability to be Parent and Care for a Child [9, 35, 37, 38, 42, 44, 45, 50]
		Using contraceptives or pregnancy prevention methods [6, 11, 14]
		Number of fertilizations and unintended pregnancies [11]
		Psychological/psychiatric conditions [8, 25, 32, 44, 45]
		Physical health status of the couples [8, 31, 32, 35, 38, 44, 45]
		Availability of health promotion programs [12]
**Societal and Cultural Context**	**Socio-Cultural Factors**	Impact of Social Networks, Virtual Space, and Mass Media [23, 33, 38, 41, 43, 51]
		Cultural/Religious Values and Beliefs [8, 26, 37, 38, 42–46, 49, 50, 52]
		Elevating the Quality of Social and Cultural Relations [12]
		Implementation of Educational Programs on Marital Life Skills for Youth and Enhancement of their Perspective Towards Parenthood [42]
		Social Imitation/Social Learning [4, 33, 37, 38, 41, 46, 49, 51]
		Addiction and Criminality Among Women [49]
		Housing Situation (Owned or Rented) [8, 12, 44, 45, 49, 51]
		Residence Location (Urban or Rural) [8, 10, 15, 17, 40, 42, 43, 52]
	**Social and Institutional Support**	Parent’s Employment/Unemployment and Job Security [2, 4, 5, 40, 42]
		Concerns about Children's Job Security [42, 45]
		Spouses’ Occupation type [12, 14, 17, 30, 42] and Their Job Conditions [4, 8, 35, 36, 38]
		Availability of Child Care (Kindergarten) [5, 35]
		Social Support [4, 12, 26, 42, 43, 46] and Social Security [49]
		Non-reduction of wages during maternity leave [36]
		Receiving pregnancy, breastfeeding, and childcare assistance related expenses from the workplace [36]
		Availability of facilities suitable for pregnancy and breastfeeding in the workplace [36]
		Presence of schools and daycare centers near the workplace [36]
		Presence of family counselors, parenting classes, and medical counselors and assistants during pregnancy at the workplace [36]
		Availability of transportation services during pregnancy and breastfeeding at the workplace [36]
		Availability of rest areas during pregnancy and breastfeeding at the workplace [36]
		Alignment and adjustment of daycare center hours with regular working hours [36]
		Reduction of working hours for pregnant mothers [36]
		Preservation of job position after maternity leave [36]
		Performing specific tasks during pregnancy and breastfeeding remotely and through telecommuting [36]
		Creating favorable working conditions during and after pregnancy [42]
		Creating conditions for maternal role compatibility with women's education after marriage [42]
**Economic Landscape**	**Economic and Financial Factors**	Financial Concerns [6, 37]
		Household Economic Status or Income Level [1, 5, 7, 8, 10, 13, 15, 23–25, 29, 31, 34–36, 42–47, 49–52]
		Infertility Treatment Expenses [42]
		Childbirth Expenses [1, 32, 37–39, 42, 44, 45, 50, 51] and Healthcare and Medical Costs [7, 16]
		Insurance Policies/Health Insurance [12]
	**Government and Policy Interventions**	Limitations on providing family planning services at health centers [49]
		Improving working conditions for women [42] and Legal support for employed women [36, 42]
		Exceptional Insurance Support for Infertile Couples [42]
		Free Maternal and Child Insurance Coverage [42]
		Retirement Insurance for Stay-at-Home Women [10]
		Social Support for Women's Childbearing through the Insurance System [42]
		Government support in obligating employers to adhere to laws benefiting pregnant and mother women in the workplace [3, 36, 51]
		Providing cash subsidies [42, 45]
		Housing allocation [3, 42]
		Providing interest-free loans and housing assistance [42]
		Providing gold coins [42]
		Monthly free check-ups and free food baskets for pregnant mothers [10, 42]
		Tax discounts based on the number of family children [42]
		Increasing work experience for employed mothers per child [42]
		Extending the legal age for education and obtaining housing deposit loans from universities [42]
		Increasing maternity leave for employed or studying mothers [36, 42, 45, 49, 52]
		Granting paternity leave after childbirth [42, 49, 52]

6. Consultation with experts

This step is optional in some scoping review guidelines, but in the framework provided by Levac [19], getting experts’ opinions regarding the obtained items through consultation is emphasized. Therefore, it tried to use some experts’ suggested viewpoints in this field to finalize the research findings.

## Results

Through a meticulous search, 5,346 studies were identified across 3 primary databases. After eliminating duplicates, 2,756 studies proceeded to the title screening phase. Subsequently, studies with irrelevant titles were excluded, leaving 1,134 studies for abstract review. From this, 336 studies were identified as suitable for full-text assessment. Also, 23 studies were added to this screening phase due to experts’ suggestion or by checking the references’ lists. After a comprehensive evaluation of the complete texts, 46 articles met the criteria for inclusion in the scoping review and subsequent data extraction.

The majority of the studies were conducted in 2021 (19.5%), followed by 2017 (15%), 2022 (13%), and 2019 (13%). The predominant focus of the final studies derived from the scoping review was Iran (65%). However, within the scoping review, four selected studies explored factors influencing couples’ fertility preferences across a range of countries spanning from three to seventy-nine nations. Additionally, the selected studies in the scoping review employed diverse methodological approaches: cross-sectional and observational studies constituted 78%, qualitative studies comprised 11%, and systematic reviews constituted 9%. An overview of the chosen final studies based on the systematic scoping review is presented in Table 2.

Also, Table 3 presents the results of the quality assessment of the final studies derived from the scoping review, utilizing various quality assessment tools categorized by study type.

Through qualitative data analysis extracted from the selected studies in the systematic scoping review, factors influencing households’ fertility preferences were categorized into 8 primary themes and 101 sub-themes. The primary themes about factors affecting households’ fertility preferences encompassed individual factors, demographic and familial factors, cultural factors, social factors, health-related factors, economic factors, insurance-related factors, and issues related to governmental support and encouragement policies (as detailed in Table 4).

## Discussion

Based on the findings of the present study conducted through a systematic review framework, influential factors affecting couples’ inclination towards parenthood have been grouped into eight domains: individual factors, demographic and familial factors, cultural factors, social factors, health-related factors, economic factors, insurance-related factors, and government policies supporting or encouraging parenthood.

### Personal- family related factors

Age is a significant factor that profoundly influences couples’ desire for parenthood. Bagheri and Saadati (2019) investigated the dynamics of parenthood aspirations, concluding that age plays a pivotal role in shaping this inclination. Specifically, women between the ages of 20 to 39 exhibited a higher tendency toward fertility [28]. Vahdani et al. (2018) also asserted that marriage and maternal age influence fertility patterns [52]. This phenomenon can be interpreted as advanced maternal age potentially leading to medical risks for both the mother and child or as women’s fertility potential decreases as they grow older.

Another crucial determinant of couples’ fertility aspirations is their level of education. Attaining an appropriate education level, securing stable employment, and maintaining a good income are noteworthy factors shaping parental decisions regarding parenthood. A study by Araban et al. (2020) demonstrated that increased education and employment among women led to diminished fertility intentions [4]. Similarly, Rahman et al. (2020) highlighted that higher education levels correlate with reduced fertility rates for both genders [16].

Satisfaction within marital life and the quality of the spousal relationship are critical influencers of fertility aspirations. An analysis by Kariman et al. (2016) on the determinants of initial fertility choices established that making decisions about childbearing is influenced by multifaceted factors. These encompass personal aspects such as the age of marriage, optimism, quality of life, family-related factors like marital contentment, and social aspects such as social support [29]. Correspondingly, Ayazi et al. (2021) underscored that variables like higher education levels, women’s social involvement, and dissatisfaction with marriage are notably linked to women’s reservations about embracing motherhood [46].

Capacity for nurturing and competence in childcare are critical factors shaping couples’ readiness for parenthood. Ramezankhani et al. (2013) emphasized in their study that parenthood is a conscious decision rather than an arbitrary event. The decision-making process revolves around addressing specific needs. When couples feel confident in managing circumstances and raising a child, the intent for parenthood becomes evident [35]. Furthermore, concerns about their offspring’s future significantly impact couples’ perspectives on having children. In the investigation of factors contributing to reduced fertility interest among married Iranian adults, Haerimehrizi et al. (2017) identified primary factors as worries about the child’s future (76.1%) and economic challenges (71.0%) [39].

One of the significant influences on couples’ inclination towards parenthood is the current number of children they have. Ahinkorah and colleagues (2021) conducted a study demonstrating that couples with more children tend to have lower levels of desire and motivation for parenthood than couples with fewer or no children [15]. Evens and colleagues (2015) also identified influential factors on pregnancy intentions in South Africa and Malawi, concluding that spouses’ expectations for pregnancy, financial concerns, current family composition, and experiences with pregnancy prevention are crucial factors affecting the desire for parenthood [6].

Another notable factor is the support of parents, especially the spouse, in child-rearing. A study by Moradi and colleagues (2017) examined factors related to parenthood intentions, revealing that various elements contribute to these intentions. These factors include personal considerations, familial dynamics, the spouse’s role, social support, beliefs, and financial aspects. Among these factors, familial dynamics and spousal support significantly enhance women’s intentions toward parenthood [26].

### Cultural and social factors

Cultural factors, religious and national values, and beliefs substantially shape attitudes toward fertility. Rahman and colleagues (2022) conducted a study that concluded that factors like social status, societal culture, urban or rural residence, educational level, values, religious beliefs, and ethnic affiliations influence couples’ reproductive decision-making [42]. In another study, Vahdani and colleagues (2018) demonstrated that religious beliefs positively impact fertility growth. Conversely, low economic status, high literacy rates, and cultural activities negatively correlate with childbearing [52].

Ghaffari and colleagues (2021) conducted a qualitative study examining influential factors on reproduction from the perspective of women. They found that the fear of social insecurity reduces the desire for fertility. However, cultural and religious factors contribute to an increase in childbearing. Additionally, governmental encouragement and restrictive policies to enhance fertility have proven effective in promoting reproduction [49].

The inclination of couples towards parenthood is significantly influenced by their living situation, whether urban or rural. In the study conducted by Bagheri et al. (2017), Poisson regression was employed to identify preferred fertility factors, revealing that variables such as the current number of children, employment status, education level, type of marriage, and place of residence impact the desire for fertility. Notably, individuals residing in rural areas demonstrated a greater propensity toward parenthood [40]. Another pivotal social factor that plays a role in this regard is couples’ housing status. Hashemzadeh et al. (2021), in a study exploring parenthood intentions and associated factors, concluded that variables like occupational characteristics, urban residence, and housing status considerably affect fertility rates [8].

Furthermore, the influence of social networks, virtual spaces, and mass media on attitudes toward parenthood is noteworthy. Abbasi et al. (2022) demonstrated that weak cultural investment, socioeconomic status, and virtual networks, particularly the Internet, negatively impact attitudes toward parenthood [43].

### Health related factors

The physical health status of couples stands as an influential health factor affecting their inclination toward parenthood. In a study by Boivin and colleagues (2018) aimed at understanding factors preparing individuals for conception, it was reported that among considerations such as parents’ social status, economic prerequisites, personal readiness, and relational aspects, couples’ reproductive decisions are significantly influenced by their physical health. Specifically, individuals with poor health or certain medical conditions tend to lack a desire for pregnancy [31].

### Economic and insurance-related factors

One of the critical factors highlighted as a fundamental determinant of couples’ inclination toward parenthood in various studies is the economic status or household income level. Tavousi and colleagues (2016) demonstrated in their study that concerns about future economic conditions and the national economic status are among the most significant reasons for the reluctance toward parenthood [44]. In another study, Torkian Valashani and colleagues (2019) articulated that preventive reasons for refraining from parenthood encompass concerns about future education, employment circumstances, economic challenges stemming from raising new children, and insufficient income [45]. Furthermore, Wei and colleagues (2018) illustrated in their study that the financial costs of having children have a significant and negative correlation with desirable fertility, given the lost income of women who opt for motherhood and the accessible social security benefits for retired rural residents [7].

Another influential factor in the inclination toward childbearing is insurance-related issues, such as specific insurance support for infertile couples, free insurance coverage for the mother and her child, and social support for women, as mentioned in a study by Rahman and colleagues (2022) [42]. These supportive policies can create motivation and a greater willingness for couples to have children, consequently contributing to population growth.

### Government incentive policies: a catalyst for population growth

One of the most pivotal and influential factors affecting population growth and households’ propensity towards childbearing is the strategic implementation of effective and meticulously planned government incentive policies. The essence of these policies lies in cultivating the desire for childbearing among couples, as elucidated by a study conducted by Samani et al. in 2020. This study investigated the impact of legal support for employed women during pregnancy and breastfeeding on their inclination toward having children. The conclusive outcome highlighted a substantial and positive correlation between legal support and employed women’s fertility inclinations, underscoring the imperative for such forms of support to be structured to amplify the motivation for childbearing [36].

## Conclusion

Drawing from the insights of this research, it becomes evident that governments and policymakers must bestow comprehensive and well-directed attention to the myriad of factors influencing households’ intentions and behaviors related to childbearing. Furthermore, given the comparatively limited effectiveness of specific existing government incentive policies in stimulating couples’ aspirations for childbearing, a reassessment and reformulation of these policies seem indispensable. Particularly crucial is addressing paramount challenges and factors contributing to couples’ concerns about childbearing or reinforcing elements that could significantly enhance fertility desires and intentions for childbearing.

## Data Availability

The datasets used and/or analysed during the current study are available from the corresponding author on reasonable request.

## Abbreviations

PCC: Population, concept, and context
PRISMA: Preferred Reporting Items for Systematic Reviews and Meta-Analyses
